# Management dilemma in choosing evolving treatments in neutropenic septic shock

**DOI:** 10.1007/s00467-025-06798-y

**Published:** 2025-06-21

**Authors:** H. David Humes, Kera Luckritz, Stephen Gorga, Katie Plomaritas, Sara Hoatlin, Michael Humes, Lenar Yessayan

**Affiliations:** 1 Department of Internal Medicine (Nephrology), University of Michigan, Ann Arbor, MI USA; 2 Innovative Biotherapies, Ann Arbor, MI USA; 3 Department of Pediatrics (Nephrology), University of Michigan, Ann Arbor, MI USA; 4 Department of Pediatrics (Critical Care), University of Michigan, Ann Arbor, MI USA; 5 Clinical Nursing Service (Pediatrics), C.S. Mott Children’s Hospital, University of Michigan Medical Center, Ann Arbor, MI USA

**Keywords:** Sepsis, Neutrophils, Neutropenia, Shock, Immunomodulation, Extracorporeal device

## Abstract

How does a physician decide to use a recently FDA-approved life-saving device in a desperately ill child in which little prior clinical experience is available? This report presents a pediatric patient with neutropenic septic shock and multiorgan failure (MOF) with a 95% chance of death and the availability of a therapeutic device with a completely new approach to treat sepsis. This device, called the selective cytopheretic device (SCD), is a first-in-class autologous immune cell directed therapy. The SCD, when integrated into an extracorporeal blood circuit, has been shown to bind activated neutrophils and monocytes. With a simple pharmacologic maneuver within the device, the bound cells in real time are immunomodulated from a highly pro-inflammatory state to a less inflammatory phenotype. These transformed cells are then released back into the systemic circulation thereby tempering the systemic hyperinflammatory disorder. Since this cell directed therapy focuses on neutrophils, the processing of these cells in a neutropenic state may be a substantive risk resulting in further immunosuppression. On the other hand, the immunomodulation of the circulating neutrophils and monocytes, although sparse, may be beneficial to disrupt the dysregulated inflammatory state responsible for ongoing tissue damage and organ dysfunction. Prior clinical SCD trials excluded patients with neutropenia so that no prior clinical experience was available to make a difficult decision. This report presents the way the medical team approached these issues and made a therapeutic plan that resulted in a positive clinical outcome for the patient.

## Case report

A 10-year-old male with history of living donor kidney transplant at the age of one year for chronic kidney disease (CKD) stage 5 secondary to posterior urethral valves was admitted to the hospital for evaluation of a new abdominal mass. At the time of admission, he was on triple immunosuppression with tacrolimus, mycophenolate, and prednisone. He was found to have leukocytosis, elevated LDH, and uric acid levels with concern for post-transplant lymphoproliferative disorder (PTLD). A PET scan demonstrated a large epigastric mass with lymph node, hepatic, and diffuse bone involvement. A measurement of Epstein Barr virus (EBV) was greater than 4.5 million I.U./mL plasma DNA. A bone marrow biopsy demonstrated mature B-cell leukemia Burkitt-type. Mycophenolate was discontinued.

Induction chemotherapy was initiated. Five days later, MRI demonstrated a decrease in size of the epigastric mass and liver lesions. He was discharged from the hospital after 17 days. Two days later he returned to clinic for further chemotherapy. At that time, he was neutropenic with a WBC of 200 cells/µL. Two days later, he returned to the emergency room presenting with fever, weakness, abdominal pain, and bloody diarrhea. Upon evaluation, he was febrile, tachycardic, hypotensive, hypoxic, and jaundiced. Blood laboratory parameters revealed pancytopenia: WBC < 100 cells/µL, hemoglobin 8.5 g/dL, platelet count 2 K/µL; electrolytes (mmol/L): sodium 115, potassium 4.7, chloride 84, and bicarbonate 17; BUN 74 mg/dL, creatinine 2.68 mg/dL; lactate 6.1 mmol/L; AST 70 U/L, ALT 611 U/L, and bilirubin 11.1 mg/dL; and procalcitonin > 100 ng/mL, prothrombin time 23.3 s., INR 2.3, and PTT 60.8 s. Rhinovirus and RSV were detected by nasal respiratory viral panel. Blood cultures were drawn and were eventually positive for *Pseudomonas aeruginosa*. He was diagnosed with septic shock with multiorgan failure (MOF) and disseminated intravascular coagulation. He was admitted to the PICU. He was volume resuscitated, placed on supplemental oxygen, and was started on epinephrine, norepinephrine and vasopressin as well as multiple broad spectrum antibiotics and stress-dosed hydrocortisone. His kidney function worsened with concurrent oliguria prompting initiation of continuous kidney replacement therapy (CKRT). His trachea was intubated, and he was placed on mechanical ventilation. He received multiple platelet and blood transfusions. Possible thrombocytopenia-associated (TA)MOF was entertained with a low ADAMTS13 activity reported as 0.35 IU/mL (nl: > 0.60 IU/mL). Anakinra was started on hospital admission day (HAD) 2 and continued until HAD 18. He stabilized but with his multiorgan failure, including cardiovascular, respiratory, kidney, liver, and hematologic failures; his risk of mortality was 95% based on Pediatric Risk of Mortality (PRISM) III score of 22 upon ICU admission.

## Management dilemma

Accordingly, therapies targeting the excessive host response to sepsis needed to be considered. Therapeutic plasma exchange (TPE) was considered as an established intervention in pediatric TAMOF. A recently FDA-approved immunomodulatory device, called the Selective Cytopheretic Device (SCD) was also considered. The SCD (brand name QUELimmune) is an autologous immune cell directed device demonstrated to temper the hyperinflammatory state of multiple disease processes, including sepsis. The medical staff was familiar with its use since it was invented at the University of Michigan and multiple adult and pediatric trials of the device had been conducted at this site. The difficulty was the FDA indication excluded neutropenia due to SCD’s unknown risk to immunomodulate neutrophils and monocytes in the presence of leukopenia. Due to the desperate situation, the medical staff was able to obtain emergency use approval from the local Institutional Review Board (IRB) to undertake SCD therapy.

**Fig. 1:**
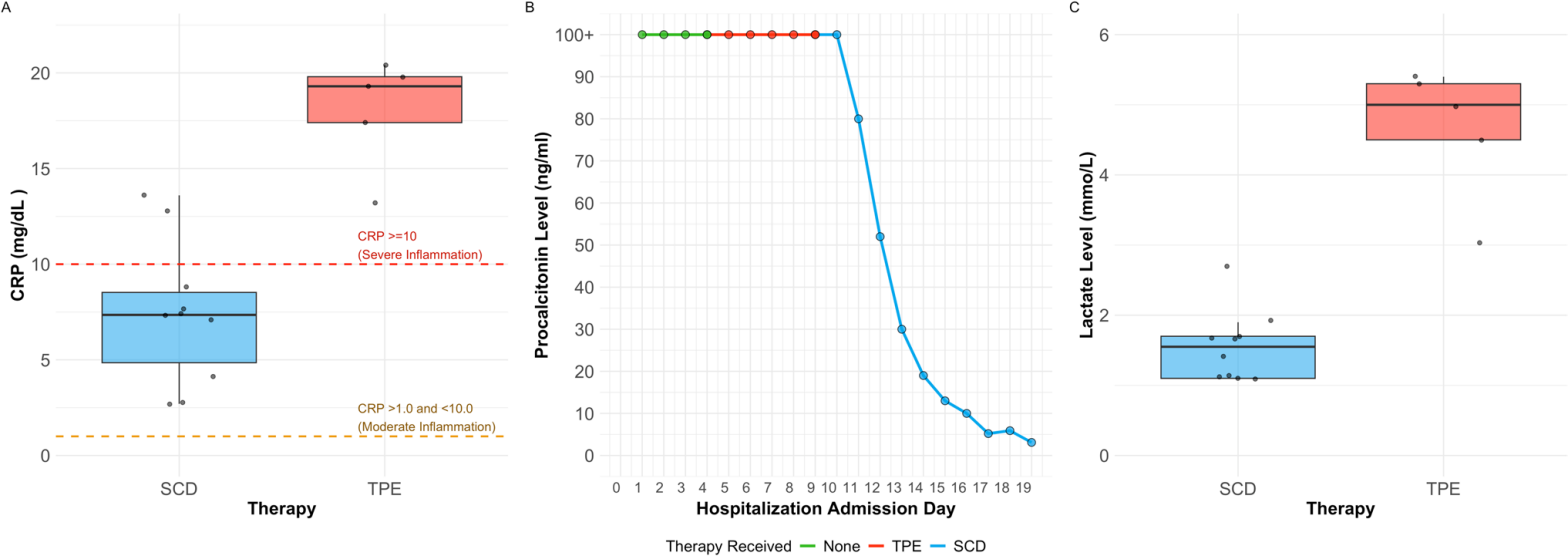
Changes in CRP, procalcitonin, and lactate levels during TPE or SCD treatments. **A** Boxplot overlaid with jitter plots of daily CRP levels (mg/dL) by type of therapy. The boxes extend from the 25 th to the 75 th percentile and are bisected by the median; the whiskers extend to the most extreme value within 1.5 of the interquartile range. A significant difference in CRP levels was found by a paired *t*-test between days with SCD and TPE therapy (*p* < 0.001). **B** Trendline of procalcitonin levels (ng/mL) by hospitalization admission day and type of therapy. Procalcitonin levels for days with TPE treatment remained at > 100 whereas procalcitonin levels on days with SCD therapy showed steady decline and an overall mean of 31.8. **C** Boxplot overlaid with jitter plots of daily lactate levels (mmo/L) by type of therapy. The boxes extend from the 25 th to the 75 th percentile and are bisected by the median; the whiskers extend to the most extreme value within 1.5 of the interquartile range. A significant difference in lactate levels was found by a paired *t*-test between days with SCD and TPE therapy (*p* < 0.001)

After discussions among the medical staff, a plan was formulated to evaluate SCD therapy on the patient for one 24-h treatment for a risk/benefit assessment followed by the more conventional TPE treatment. If the patient was still critically ill after TPE, and the first SCD treatment was tolerated without serious adverse events in the face of neutropenia and thrombocytopenia, a full 10-day treatment plan with the SCD would be initiated. As detailed in Table 1, this interventional plan was successful to reverse the acute organ dysfunction and hyperinflammatory state of the patient. The first SCD treatment occurred on the third hospitalization admission day (HAD). No adverse events were observed. As planned, five subsequent 1.5 × plasma exchange volume TPE treatments were undertaken. During TPE treatments his vasoactive need decreased but his inflammatory indices continued to be highly elevated, and his lactic acidosis persisted. SCD treatments were then re-initiated on HAD 9 and treatment with the SCD was associated with declines in C-reactive protein (CRP) and procalcitonin along with resolution of his lactic acidosis. He no longer required vasoactive medications (Fig. 1). In fact, the hypotension resolved while on SCD therapy to the point of requiring nicardipine for control of hypertension and his stress-dosed hydrocortisone was weaned. His liver function tests also normalized with ALT 47 U/L, AST 57 U/L, and total bilirubin 1.1 mg/dL. His pancytopenia began to resolve on HAD 5 during SCD therapy and his neutrophil count was normal at the end of SCD treatment. Of importance, evaluation of the SCD device after the first 24-h treatment demonstrated binding of 10^6^ highly activated neutrophils while the absolute circulating neutrophil count was less than 100/μL. As his inflammation resolved, net volume removal with CKRT was able to safely remove volume during SCD therapy resulting in less pulmonary edema and improved respiratory function so that on HAD 25 his trachea was extubated and supported with CPAP. His urine output also improved to greater than 2 L per 24 h; he was taken off CKRT on HAD 25. During this period, he had other complications including evidence early on in his course of neutropenic colitis and a persistent intra-abdominal fluid collection. This collection was drained and grew *Pseudomonas aeruginosa* suggestive of perforation. Due to his critical condition and thrombocytopenia, surgery service elected to follow and treat with antibiotics. On HAD18 blood cultures from the hemodialysis catheter were positive for methicillin resistant *Staphylococcus epidermidis*; he was started on vancomycin and later daptomycin.

**Table 1:**
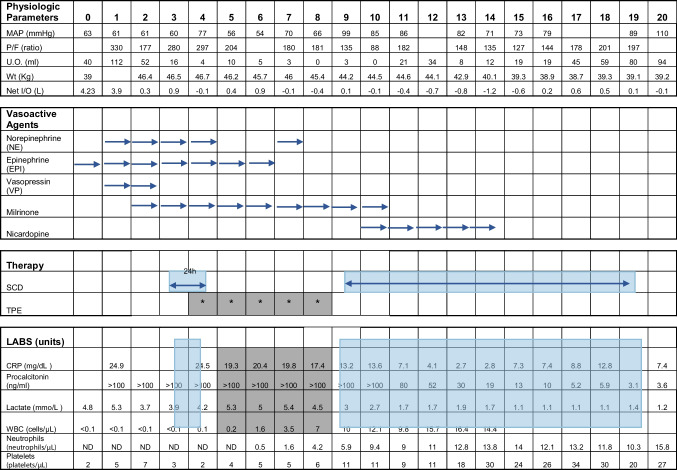
Physiologic parameters, vasoactive agents, therapy and laboratory tests by hospital admission day (HAD)

The day after extubation and CKRT discontinuation, the patient had an acute decline in his hemoglobin and imaging procedures demonstrated evidence of ileal perforation and air in the intrabdominal fluid collection. This development was most likely due to his evolving neutropenic enterocolitis. He was urgently taken to the operating suite where he underwent removal of cecum, terminal ileum and appendix. He returned to his ICU bed with an open surgical wound and was re-intubated, and CKRT was re-initiated. Several days later after additional surgery for ileostomy creation and wound closure, he was once again extubated successfully, and his kidney function recovered such that he no longer required dialysis. He did not require kidney replacement therapy since HAD 35. After 38 days in the PICU, he was transferred to the general pediatric ward. He continued to improve and initiated his second course of chemotherapy on HAD 54. Bone marrow biopsy three weeks later showed no evidence of lymphoma, and he was discharged home on HAD 73.

## Discussion

This patient presented with neutropenic sepsis. He was rapidly deteriorating with evidence of 5 organ system failures: cardiovascular, respiratory, kidney, liver, and hematologic. The development of sepsis was an anticipated complication of the patient undergoing intensive cytotoxic chemotherapy [1]. His rapid progression to septic shock and MOF portended a grave prognosis with a PRISM III score predicting a 95% chance of mortality. Following pediatric Surviving Sepsis Campaign guidelines (2) broad spectrum antibiotic coverage, volume resuscitation and stress steroids were administered. As he progressed to septic shock and organ dysfunction, he was started on multiple vasoactive medications, mechanical ventilation and CKRT. Unfortunately, he continued to deteriorate. Less established interventions including cytokine inhibitors, in this case anakinra (a recombinant monoclonal antibody to IL-1 receptor antagonist [3]), were initiated, and TPE was considered to reduce the levels of the soluble mediators of the cytokine storm. Also entering the discussion was the use of a recently FDA-approved immunomodulatory therapeutic device, called the Selective Cytopheretic Device (SCD) to treat pediatric sepsis [4]. According to recent Surviving Sepsis Guidelines in Children, the use of TPE to treat septic shock in children has not been established. A recent meta-analysis demonstrated that TPE was associated with reduced mortality in adults but not children, although a subgroup analysis in children with TAMOF demonstrated a clinical benefit [5]. Similarly, FDA HDE approval of SCD therapy was based on safety and probable benefit of the device in children. Recent consensus statements from the European Pediatric Intensive Care Units [6] acknowledge the SCD as a potential adjunctive extracorporeal blood treatment for septic shock.

### New evolving innovative technology

The SCD is an extracorporeal autologous immune cell directed device and targets the circulating cells of the innate immunologic system in disorders of hyperinflammation. This approach focuses on the underlying cause of progressive life-threatening organ dysfunction promoted by sepsis. Tissue injury and organ dysfunction in sepsis evolves primarily due to activated circulating neutrophils interacting with activated tissue [7]. This interaction results in sludging and obstruction in capillary beds and poor tissue perfusion with ischemic consequences to vital organs. The interaction of activated neutrophils and endothelium also leads to increased vascular permeability with fluid leakage from the intravascular space to tissue interstitium with resulting hypovolemia, hypotension, and shock requiring vasoactive medication support. The increased permeability of the endothelium also allows neutrophil and monocyte migration into interstitial spaces with subsequent release of degradative enzymes and destructive moieties resulting in further tissue injury. The combination of ischemic and toxic injury leads to multiorgan dysfunction and failure. Activated neutrophils and monocytes are thus central players in the pathophysiology of sepsis-induced life threatening organ failure.

**Fig. 2:**
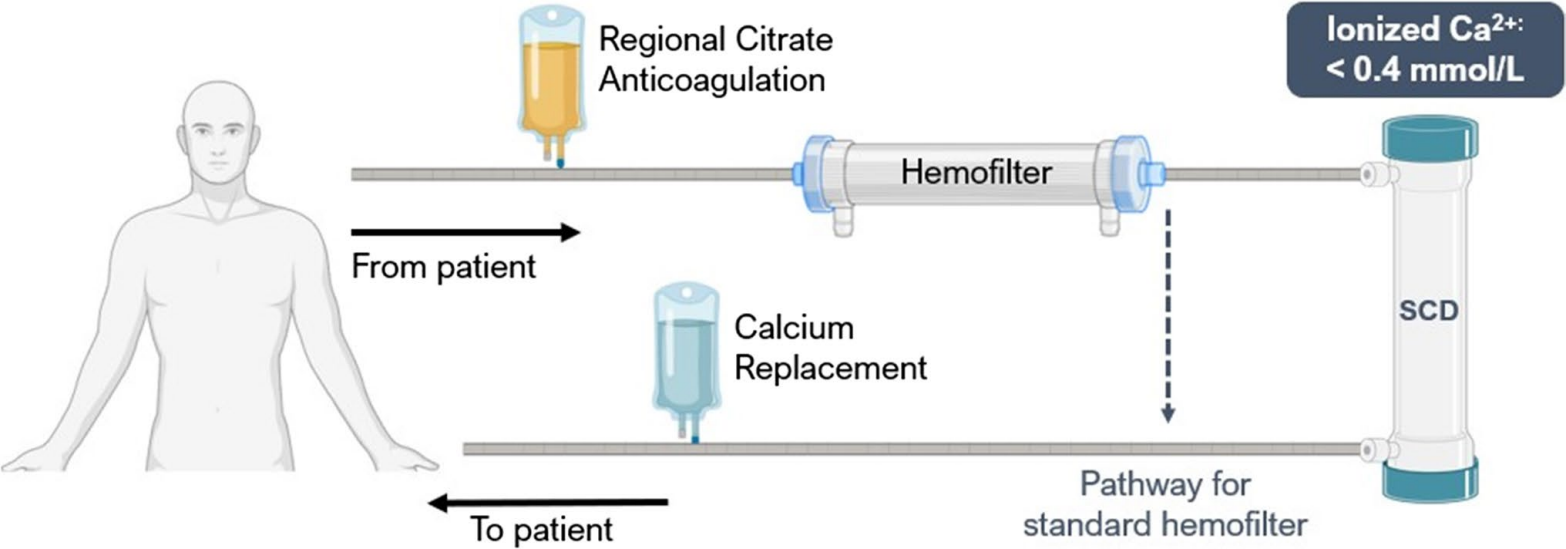
Extracorporeal blood circuit for SCD treatment

The SCD is composed of a polycarbonate cylindrical housing containing fibers made of synthetic biocompatible, biomimetic membranes with a total surface area depending on therapeutic dose (Fig. 2). Therapeutic administration is delivered via an extracorporeal blood circuit and is delivered continuously with a new device exchanged every 24 h. The design of the cartridge incorporates a low-velocity, low-shear force blood flow path around the bundled, biocompatible fibers. Due to the low shear force, approximating capillary shear, activated neutrophils bind and are sequestered for up to several hours along the membrane surface. The sequestered, activated neutrophils are then immunomodulated when exposed to a low ionized calcium (iCa) environment (0.25–0.4 mM) afforded by clinical protocols for regional citrate anticoagulation (RCA) of the blood passing through the SCD [8, 9]. Maintenance of a low iCa level in the SCD is critical not only to maintain anticoagulation within the circuit but also to provide the immunomodulation activity of the device [10]. The low iCa environment within the extra-capillary space of the device promotes the selective binding of the most activated circulating neutrophils and monocytes in the blood cell surface molecules of these leukocytes requiring calcium to bind to surfaces [11, 12]. Lymphocytes are not bound. The more activated the cells, the more binding molecules are expressed on the cell surface, thereby increasing the density of these binding sites on the cell and causing more intense binding to the membranes. Exposure of these cells in the low iCa environment within the SCD results in release of these cells back to the patient with a less proinflammatory phenotype resulting in a dampening of the overall activated state of the circulating population of neutrophils. More specifically, recent data demonstrates that cell processing of neutrophils within the device results in the initiation of the apoptotic program of the bound neutrophils [10], as has been previously reported in low iCa environments [13, 14]. The neutrophils are then released back into the circulation where the normal clearance of apoptotic neutrophils occurs via phagocytosis and digestion by macrophages within the bone marrow, spleen, and liver [15, 16]. The cell processing is, therefore, selective for the most activated circulating neutrophils and the subsequent release of these bound neutrophils to cell senescence provides a natural clearance process of the damaging circulating cells within the body. Immunomodulation rather than immunosuppression or immunodepletion is achieved. This process of catch, immunomodulation and release has been confirmed in multiple in vitro bench testing and clinical evaluations. This continuous neutrophil processing within the SCD is an elegant manner to temper the excessive, dysregulated hyper-inflamed state and allows tissue repair and recovery to occur.

The SCD may also play a role in the repair and recovery of chronic organ dysfunction as well as acute organ failure due to its effects on circulating monocytes. Circulating monocytes play a critical role in the repair of damaged tissue as well as progression of chronic organ dysfunction following acute insults [17]. The migration of circulating monocytes into tissue during injury leads to a transformation into tissue macrophages. The phenotype of the circulating monocyte and the local tissue environment determines whether the monocyte transitions to a degradative (M1) or a reparative (M2) phenotype. The SCD has been shown to bind the most inflammatory circulating monocytes. During the sequestration within the SCD, the monocytes are transformed due to changes in gene expression (studies are ongoing utilizing single cell transcriptome analysis) and released in a less inflammatory phenotype [10, 18]. This SCD effect on circulating monocytes may influence tissue macrophages as an added mechanism to suppress hyperinflammation and improve tissue repair.

Pre-clinical large animal studies have demonstrated that SCD therapy improves organ dysfunction in acute kidney injury (AKI) due to sepsis, acute lung injury, acute-on-chronic heart failure, and hemorrhagic stroke [19]. Translation into the clinic has been encouraging with a compelling safety profile [20] and strong efficacy signals in treated ICU patients with hyperinflammation suffering from AKI requiring CKRT, acute respiratory distress syndrome (ARDS) requiring mechanical ventilation, cardiorenal syndrome (CRS) and hepatorenal syndrome (HRS) [19, 21]. Since SCD treatment focuses on tempering neutrophil activation, all prior clinical trials have excluded patients with neutropenia due to uncertainty of the risk/benefit of therapy in these patients.

### Approach to this medical dilemma

With these perspectives, the medical care team was confronted with a dilemma of choosing between TPE or SCD treatments. The use of the SCD was further complicated by the lack of prior experience of the risk/benefit of this form of therapy directed specifically for activated neutrophils in the face of severe neutropenia. After considerable deliberation, the team decided on a plan to evaluate SCD therapy on the patient for one 24-h treatment for a risk/benefit assessment followed by a more conventional TPE treatment plan. If the patient was still critically ill after TPE and the first SCD treatment was tolerated without serious adverse events in the face of neutropenia and thrombocytopenia, a full 10-day treatment plan with the SCD would be initiated. As detailed in Table 1 and Fig. 1, five TPE treatments seemed to stabilize the patient but there were no major improvements in inflammatory mediators or his elevated lactate levels. After the re-initiation of SCD therapy, significant declines in inflammatory markers and lactate levels were observed. The period of treatment with TPE and SCD were compared assessing key inflammatory markers: C-reactive protein (CRP) and procalcitonin along with lactate. The CRP levels remained highly elevated during treatment with plasmapheresis averaging 19.2 mg/dL (range 17.4–20.4) while CRP levels were significantly reduced during SCD treatment with an average of 7.4 mg/dL (range 2.7–13.6). Procalcitonin remained unchanged during TPE treatment at > 100 ng/mL but steadily declined during SCD treatment, decreasing from > 100 ng/mL on the first day of treatment down to 5.9 ng/mL on the last day of treatment, with an average of 31.8 ng/mL. Lactate also showed declines, with an average of 5.1 mmo/L (range 4.5–5.4) during plasmapheresis treatment and 1.6 mmo/L (range 1.1–2.7) during SCD treatment. Differences in both CRP and lactate levels by therapy treatment type were significant by paired t-test (*p* < 0.001). The IRB also allowed evaluation of the SCD device after the first day of therapy. Cells bound to the SCD were almost all activated immature neutrophils without monocytes or lymphocytes. The cell count was approximately 1 × 10^6^ compared to prior clinical studies demonstrating greater than 5 × 10^8^ [22]. Cell surface markers by flow cytometry demonstrated that the bound neutrophils were highly activated [22]. These observations suggest despite the patient having no detectable neutrophils in the circulating blood, the SCD was able to bind a reasonable number of highly activated neutrophils and immunomodulate them resulting in a reduction in systemic inflammatory biomarkers. The temporal correlation of the decline in these biomarkers with SCD therapy are suggestive of an immunomodulatory effect of SCD therapy not observed in the prior 5 days of TPE therapy. The ability to capture highly activated circulating neutrophils suggests that despite an inability to measure neutrophils in blood samples, activated neutrophils are in fact circulating and important in the dysregulated host response responsible for tissue and organ injury. Similar rapid decline in inflammatory biomarkers after the initiation of SCD therapy in a toddler with HLH and multiorgan failure has been reported [23].

This case study presents how our group handled a medical management dilemma in the use of evolving early innovative technology which has had little prior clinical experience in a desperate clinical situation. This case summarizes the stepwise decision-making process which ultimately resulted in a favorable outcome in a desperately ill child. 

### Key management points*

**Table:** 

1	Antimicrobial therapy (*strong recommendation, very low quality of evidence*)
2	Fluid resuscitation (*weak recommendation, low quality of evidence*)
3	Vasoactive medications: epinephrine or norepinephrine rather than dopamine (*weak recommendation, low quality of evidence*)
4	Stress steroids (*weak recommendation, low quality of evidence*)
5	Anakinra: Il-1 receptor antagonist (*no recommendation*)
6	Therapeutic plasma exchange for TAMOF (*weak recommendation, very low quality of evidence*)
7	Selective cytopheretic device (*no recommendation*)

*Grade of evidence and recommendations from [2]
